# Selective effects of focusing on spatial details in episodic future thinking for self-relevant positive events

**DOI:** 10.1007/s00426-022-01668-w

**Published:** 2022-03-09

**Authors:** D. J. Hallford, S. Cheung, G. Baothman, J. Weel

**Affiliations:** 1 School of Psychology, Deakin University, 1 Gheringhap St, Geelong, VIC 3220 Australia; 2 School of Psychology, Deakin University, 221 Burwood Hwy, Burwood, Melbourne, VIC 3125 Australia

## Abstract

Mental simulations of positive future events increase their detail/vividness and plausibility, with effects on cognitive-affective processes such as anticipated and anticipatory pleasure. More recently, spatial details have been distinguished as important in increasing detail and elaborating mental scene construction. Building on this research, this study (*N* = 54; *M* age = 26.9) compared simulations of positive, self-relevant future events spatial details (i.e. people, objects, sequences of actions) with simulations focused on content details. Cross-sectionally at baseline, spatial details uniquely predicted phenomenological characteristics of future events, including anticipatory pleasure. The guided simulations increased detail and vividness, mental imagery, and pre-experiencing in both conditions. The content simulation condition did not increase content details relative to the spatial simulation condition, however, the inverse was true. Relatedly, overall detail and vividness were higher in the spatial condition, as was perceived control. The findings are discussed in relation to future thinking and mental health.

## Introduction

Episodic future thinking refers to mentally simulating self-relevant future events. It is the future-oriented version of episodic thinking that draws on and overlaps with episodic memory for past events (Schacter et al., 2007). Imagining future events has a range of functions, such as in planning and initiating goal-directed behaviour, spatial navigation, problem-solving, and emotional regulation (Schacter et al., 2017). Studies have shown that engaging in effortful simulations of future events can increase their subjective detail and vividness (e.g., Boland et al., 2018; Hallford et al. 2020d) and their plausibility (Boland et al., 2018; Gregory et al., 1982; Szpunar & Schacter, 2013). As Kahnemann and Tversky (1998) have proposed through their simulation heuristic, the more easily and realistically a future event comes to mind, the more likely it is perceived to occur (Taylor & Schneider, 1989).

Distinctions have been made between the types of details generated in episodic future thinking, and how this affects phenomenological characteristics and judgements of future events. The philosopher Immanuel Kant (1724–1804) famously proposed that representing space in one’s mind is essential to organising senses and intuitions (Smith, 2011). Pursued more recently and empirically in cognitive research, spatial details have been associated with a stronger sense of pre-experiencing and emotional resonance (Rubin & Umanath, 2015). Scene construction theories suggest that spatial features are key in generating contextualised, specific events in which contents can be organized and interpreted (e.g., Hassabis & Maguire, 2007; Rubin & Umanath, 2015; Rubin et al., 2019). This is thought to be because constructing spatial details necessitate that a person imagines locating themselves in a particular place. Where spatial details are not generated, it may be more difficult to distinguish specific scenes from semantic de-contextualised information. Given this, future thoughts that have a clearer spatial context might be more perceptually rich, and therefore more vivid and realistic, relative to decontextualized content.

Recently, Rubin et al. (2019) showed that when remembering past events spatial features (such as identifying *where* actions, objects and people were located) were much stronger predictors relative to content features (such as identifying actions, objects and people without spatial properties) in terms of subjectively reported vividness, a sense of re-living, and belief in occurrence. While this was a cross-sectional study, it indicates that spatial features predict unique variance in how scenes are mentally represented and subjectively judged. In an experimental design, Sheldon et al. (2019) showed that individuals who were primed to focus on either spatial details or temporal details (i.e., the sequence of events) subsequently reported more perceptual details for self-relevant past and future events relative to a control condition. They reasoned that perceptual details are more strongly related to pre-experiencing than semantic-type content. Some evidence was found that priming for spatial details, relative to temporal, gave rise to a richer sense of pre-experiencing, however the findings were mixed across two studies. Notably, objective ratings of details in reported events where used. Nonetheless, the findings suggest that promoting someone’s attention on to spatial details may be particularly important in giving rise to vivid and perceptually rich mental representations, and further investigation in future thinking is warranted.

If spatial details do have a relatively stronger impact on vividness and realism, then they may also differentially impact other characteristics of future thinking and subjective judgements about future events. In particular, focusing on spatial details might be one method through which to increase anticipated pleasure (the expectation of enjoyment) and anticipatory pleasure (the feeling of pleasure from pre-experiencing) for positive events, the perceived control we have over them, and our behavioural intentions. Previous research has indicated that detail and vividness is correlated with more use of mental imagery (Hallford et al., 2020b), and that both detail and vividness and mental imagery are related to higher anticipatory and anticipated pleasure (Hallford et al. 2020a), a stronger sense of perceived control (Jing et al., 2016) Trials in clinical groups (Hallford et al., 2020c, 2021) and non-clinical groups (Hallford et al., 2020d) have already shown that training in enhancing the ability to produce detail and specificity in future thinking can influence such processes. Distinguishing the effects of particular types of details would provide a more nuanced understanding of their benefit in functioning. This might help to better specify interventions that focus on episodic thinking (e.g., memory specificity training or future specificity training; Hallford et al., 2020d; Raes et al., 2009), and enhance their effect on disrupting psychopathological processes such as dampened positive affect, reduced reward response, poorer self-efficacy, and hopelessness.

## The current study

The aim of this study was to examine whether self-reported spatial details in episodic future thinking have an effect on phenomenological characteristics and subjective judgements of imagined, self-relevant future events, relative to content details. To do this, we first sought to extend on Rubin et al.’s (2019) study and test the unique contributions of spatial and content details in predicting variance in these variables (Aim 1). It was hypothesised that spatial details would be a unique predictor, whereas content details would not. We then sought to use a previously validated protocol (Hallford et al., 2020b) to experimentally test whether guided episodic future thinking tasks would differ in their effects on these variables dependent on whether they focused on content details or on spatial details (Aim 2). Guided interviews were used to focus participants on either content or spatial details, primarily as this may have some practical relevance for implementation in an applied context where the aim might be to change judgements about future events. Given that anticipated and anticipatory pleasure were the prospective emotions of interest, the study focused on positively-valenced events. It was hypothesised that guided interview conditions that focus on either content or spatial detail conditions, relative to a baseline condition without elaboration on events, would both increase overall detail/vividness, mental imagery, pre-experiencing, anticipated and anticipatory pleasure, perceive control, and behavioural intention, and that scores on these variables would be higher in the spatial details condition.

## Methods

### Design

**Fig. 1:**
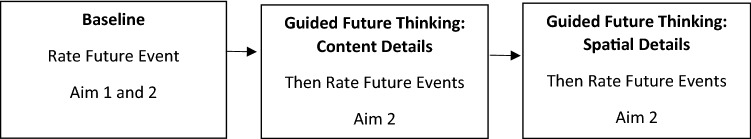
Study design. Note: Guided future thinking on content or spatial details was counterbalanced across participants

The study used a within-subjects design with a repeated measures factor of condition (baseline; content condition; spatial condition). The dependent variables, assessed in all conditions, were content details, spatial details and overall detail/vividness, pre-experiencing, the use of mental imagery, anticipated and anticipatory pleasure, perceived control, and behavioural intention. See Fig. 1 for the study design.

### Participants

The only inclusion criteria were 18 ≥ years old and English-speaking. The sample consisted of 54 participants (*M* age = 26.9, SD = 7.5, range 19–60; 66.7% female). They were recruited through advertisements on social media and snowballing. As their highest educational attainment, the majority reported having a bachelor (64.27%) or postgraduate degree (13.2%), and the remaining had a diploma or certificate (9.4%) or finished high school (13.2%). The majority identified as Caucasian/White European (45.3%), and the remaining as Asian (26.4%), Arab or Middle Eastern (15.1%), African (3.8%), or “Other ethnicity” (9.4%). Most reported being in paid employment (60.8%), and just over half were studying (56.6%).

An a priori power analysis was conducted using G*Power 3.1.9.2 (Faul et al., 2007). Based on the smallest observed effect sizes from previous work using this guided future thinking activities (Hallford et al., 2020b), namely for perceived control and behavioural intention, we powered the study to detect at least small to moderate-sized within-group effects (*d*_*z*_ = 0.40) using paired samples *t*-tests, with a power of 0.80 and alpha level of 0.05. To detect this effect, we required a total sample size of 50. We oversampled to account for some dropout, recruiting a total of 54 participants. Two of these participants completed only baseline and content details condition. To utilise all collected data, the missing responses for the spatial condition were imputed using the expectation maximisation procedure. Sensitivity analysis indicated removing these cases did not have any substantive effects on the results.

### Materials

#### Future thinking simulations

As part of the online survey at baseline, participants were then asked to select five, plausible, self-relevant future events that they were not already planning and that would be positive experiences for them. Participants were instructed they could nominate any type of event or activity and were given a range of categories as prompts: work/school/study, conversation/socializing, errands/chores, a hobby/interest, physical activity/sport, TV/internet/games/media, and ‘other’. They were instructed that the events were to occur within the space of a day and in a specific place, and some examples were given (e.g., going shopping at a local shopping mall, going for lunch with a friend at a particular restaurant). They were then asked to provide a brief description of the event. To account for temporal distance, the participants were asked to imagine the first event as occurring in the next week, the second in the next month, the third the next year, the fourth the next 5 years, and the fifth in the next 10 years. To assess the validity of this manipulation, after the event description they were asked how far into the future the event was imagined as taking place using a scale from 1 (the next 24 h) to 6 (the next 10 years). A repeated measures ANOVA comparing the mean of each of the five events on this temporal distance scale showed a clear main effect, *F*(4, 212) = 93.9, *p* < 0.001, $\eta_{{\text{p}}}^{2}$  = 0.63. Inspection of the means and follow up *t*-tests showed that each event corresponded approximately with the intended temporal distance, with significantly higher temporal distance for each subsequent future event (all *p* < 0.001): 2.0 (SD = 0.8), 2.7 (SD = 0.8), 3.3 (SD = 0.9), 4.2 (SD = 1.2), 5.1 (SD = 1.5).

To replicate methodology from Rubin et al. (2019), the same measures of content details, spatial details, and pre-experiencing were used, and rated using self-report response scales from 1 (not at all) to 7 (definitely). The content details items were, “When thinking about this future event, I can identify or name the setting where it occurs, although I might not be able to describe it clearly”, and “As I think about this future event, I can identify the actions, objects, and/or people that are involved in it, though I may not be able to say clearly where they are in relation to each other”. These were averaged together across the future events and showed acceptable internal reliability (*α* = 0.73). The spatial details items were, “When thinking of this future event, I imagined a scene in which the elements of the setting were located relative to each other in space” and “When thinking about this future event, I can describe where the actions, objects, and/or people are located in my imagination”. These were also averaged, and showed acceptable internal reliability (*α* = 0.80). The pre-experiencing items were, “While thinking about this future event, it is as if I am pre-living the event”, “While thinking of this future event, it is as if I am mentally traveling to the time and place of the occurrence”, and “While thinking of this future event, it is as if I am experiencing the feelings, emotions, and/or atmosphere that I would feel when it happens”. These were averaged across events, with acceptable internal reliability (*α* = 0.92).

The following items all used a self-report responses scale from 1 (not at all) to 9 (very much so) and were averaged across the five future events. The item, “How vivid and detailed is your thought of doing this activity?”, was used to rate overall detail and vividness (*α* = 0.74), and the item, “How much did you find yourself thinking in pictures/mental pictures about this?” to measure mental imagery (*α* = 0.61). To rate anticipated (expected pleasure from the event) and anticipatory pleasure (pleasure experienced thinking about the event), respectively, the following items were used: “How pleasant/enjoyable do you think it will be to do this activity?” (*α* = 0.66), and ‘How pleasant/enjoyable is it to just think about doing this activity?’ (*α* = 0.53). For perceived control the following item was used: ‘How much control do you think you would have over that activity occurring? As in, how easy do you think it would be to do?’ (*α* = 0.69). Lastly, to assess the intention to engage in the future events, participants were asked “How likely is it that you will do this activity?” (*α* = 0.69).

The following measures of depressive symptoms and trait anticipatory pleasure were administered at baseline in order to help describe the sample.

#### Depression, anxiety, and stress scale (DASS; Lovibond & Lovibond, 1995)

Depressive symptoms were assessed at baseline using the depression subscale of the DASS 21-item short-form. The DASS depression subscale assesses core features of depression (e.g., low mood, loss of interest, self-worth, and motivation) 7-item self-report items, and has previously shown excellent psychometric properties (Antony et al., 1998). The items are rated on a scale from 0 (did not apply to me ever) to 4 (Applied to me very much, or most of the time). Reponses are then summed, with higher scores indicating higher severity of symptoms. Internal reliability in the current sample was good (α = 0.87).

#### Temporal experience of pleasure scale (TEPS; Gard et al., 2006)

To assess trait levels of anticipatory pleasure, the anticipatory pleasure subscale of the TEPS was used. This scale uses 10 self-report items to assess the general tendency to think about positive future events and experience anticipatory pleasure. Responses were given on a 6-point scale from 1 (*Very fal*SE* for me*) to 6 (*Very true for me*), and items were summed with higher scores indicating a stronger tendency to anticipate pleasure. Internal reliability in the current sample was acceptable (*α* = 0.68).

#### Future thinking simulation protocol

The future thinking protocol was adapted from a protocol developed in our lab and used in two previous experiments to elicit detail in episodic future thinking (Hallford et al., 2020b). In this protocol, five events are generated by participants prior to any guided simulations, and then rated for various dimensions. This is used as the baseline condition. The same events are then used in the guided future thinking conditions. The participants were instructed to think of unique events that they would be personally involved in, or a unique instance of a type of event. This helped to ensure that participants used episodic future thinking, rather than just imagining event categories, referred elsewhere as personal semantics (Renoult et al., 2012). Participants were given general instructions to imagine events from a first-person perspective, as if they were actually happening, and to use mental imagery. They were asked to think only about the particular event that that was being discussed, taking place in a specific location and time, and not about experiences occurring before or after this. They were first given 30s to describe the highlight of each event and which positive emotions they would be likely to feel during that event. Then participants were questioned about the event for two minutes, in a way that was dependent on the condition: (1) about the contents of the scene without describing the environment (e.g., the people, the sequences of events, objects; e.g., “PleaSE describe some objects that appear in this event”. “Can you tell me about the order in which things will happen?”) or (2) about the spatial and environmental details (e.g., the size and shape of room or location, features of the physical space, how far apart objects in the location were from each other; e.g., “Can you tell me some details about the physical dimensions of the place in which this would happen?”, “Can you tell me about the dimensions of the place?—height, width, length?”). The full protocol is provided in the Supplementary Materials. Prompts were used flexibly dependent on what details the participants gave. A practice trial was provided at the start of each condition in which participants simulated doing a neutral task of mailing a letter to their local post office. Experimenters were trained on the simulation protocol over a series of sessions, and piloting was conducted with two participants prior to recruiting the main sample.

### Procedure

Prior to recruitment, ethical approval for the study was obtained from the University human ethics advisory group. Interested parties responded to advertisements and were followed up by Authors 2, 3, or 4. These researchers were trained on the guided future thinking protocols by the first author, and then recorded several training interviews on which feedback was given. To reduce bias in the interviewing, the protocols followed the same form and number of probing questions to elicit detail, and training feedback was aimed at effectiveness and equity across the guided future thinking conditions. All participants were followed up a researcher that had no prior relationship with them. A description of the study was given, and times were arranged to complete the two future thinking conditions over the telephone. A link to the online baseline survey was then provided at least one day prior to completing the first condition. Prior to commencing guided future thinking in both conditions, the experimenters checked with the participant that the nominated events listed at baseline were still relevant and plausible. Only one participant needed to nominate new activities, and new baseline scores for these activities were obtained prior to simulations. Participants were then guided to think of the events, dependent on the condition, with the order of conditions counterbalanced across the sample. After each simulation, the participants were asked to rate the event on the dependent variables. The participants completed their first condition an average of 3.1 days (SD = 1.8) after completing the baseline survey, and the second condition an average of 3.1 days (SD = 1.9) after the first. No compensation was provided to participants.

### Data analysis strategy

Mean scores and standard deviations were calculated for all study variables. Pearson correlation coefficients were used to test zero-order associations between the variables at baseline. For Aim 1, to assess the unique contribution of content and spatial details with the dependent variables, a series of multiple regressions were conducted. The variables of temporal distance, age, and gender were also entered as predictors, as per Rubin et al. (2019). For Aim 2, to assess for changes in the dependent variables over the future thinking conditions, repeated measures ANOVAs were conducted, with planned paired samples *t*-tests in accordance with the hypotheses, to compare differences between conditions where an omnibus effect was found. Cohen’s *d*_*z*_ was used to indicate the magnitude of effect between conditions.

In order of frequency, future events relating to work/school/study were most common (21.9%) along with ‘other activities not listed above’ (21.9%). Following these were conversation/socialising (17.8%), tv/internet/games/media (11.5%), physical activity (10.7%), eating/drinking (7%), a hobby (not physical activity; 4.8%), and errand/chores (4.4%). Full descriptive statistics for the variables are found in Table 1, and correlations between baseline variables in Table 2. Regarding the correlations, depressive symptoms were correlated with lower perceived control. Trait anticipatory pleasure (TEPS-A) was correlated with higher spatial and content details, pre-experiencing, and perceived control. All detail-related variables correlated with one another and with pre-experiencing and mental imagery. Spatial details were correlated with anticipatory pleasure and overall detail/vividness, but not anticipated pleasure, and content details were only related to overall detail/vividness. Perceived control correlated with all detail variables, but not pre-experiencing or mental imagery. Behavioural intention correlated with perceived control, but no other variables.

**Table 1:** Means and standard deviations of study variables in each condition

Measure	Baseline	Content condition	Spatial condition
DASS depression		–	–
TEPS-A		–	–
Spatial details	5.0 (1.0)	5.6 (0.9)	5.9 (0.7)
Content details	5.2 (0.9)	5.7 (0.9)	5.7 (0.8)
Overall detail/vividness	6.4 (1.4)	7.0 (1.2)	7.3 (0.9)
Pre-experiencing	4.5 (1.2)	5.1 (1.1)	5.2 (1.1)
Mental Imagery	6.6 (1.3)	7.3 (1.1)	7.6 (1.0)
Anticipated pleasure	7.7 (1.0)	7.5 (0.8)	7.5 (0.9)
Anticipatory pleasure	7.1 (1.1)	6.9 (1.0)	6.9 (1.1)
Perceived control	5.9 (1.5)	6.3 (1.1)	6.8 (1.0)
Behavioural intention	7.1 (1.1)	7.0 (1.0)	7.1 (0.9)

*DASS* Depression, Anxiety and Stress Scale, *TEPS-A* Temporal Experience of Pleasure Scale, Anticipatory Pleasure Subscale

**Table 2:** Zero-order correlations between study variables at baseline (*N* = 54)

	1.DASS depression	2. TEPS-A	3. Spatial details	4. Content details	5. Overall detail/vividness	6. Mental Imagery	7. Pre-experiencing	8. Anticipated Pleasure	9. Anticipatory Pleasure	10. Perceived Control	11. Behavioural Intention
1.DASS depression	–										
2. TEPS-A	– 0.09	–									
3. Spatial details	– 0.05	0.38**	–								
4. Content details	– 0.24†	0.34*	0.62***	–							
5. Overall detail/vividness	– 0.13	0.23†	0.59***	0.38**	–						
6. Mental Imagery	– 0.11	0.21	0.48***	0.31*	0.77***	–					
7. Pre-experiencing	– 0.04	0.39**	0.59***	0.39**	0.65***	0.55***	–				
8. Anticipated pleasure	– 0.03	0.01	0.16	– 0.07	0.46***	0.34*	0.13	–			
9. Anticipatory pleasure	0.08	0.22	0.34*	0.13	0.62***	0.58***	0.47***	0.71***	–		
10. Perceived control	– 0.31*	0.32*	0.37**	0.38**	0.32*	0.22	0.23†	0.16	0.22	–	
11. Behavioural intention	– 0.16	0.09	0.18	0.21	0.09	0.01	0.01	– 0.18	– 0.10	0.50***	–

*DASS* Depression, Anxiety, and Stress Scale, *TEPS-A* Temporal Experience of Pleasure Scale-Anticipatory Subscale

^†^*p* < 0.10, *p < 0.05, **p < 0.01, *** *p* < 0.001

### Aim 1: cross-sectional analyses of content and spatial details replicating Rubin et al. (2019)

The results from a series of multiple regressions using content and spatial details as independent variables indicated that spatial details, but not content details, were a significant predictor of overall detail/vividness, mental imagery, pre-experiencing, and anticipated and anticipatory pleasure (all results in Table 3). For pre-experiencing, gender was also a significant predictor, indicating that being female predicted stronger pre-experiencing. Temporal distance was the only unique predictor of perceived control and behavioural intention, indicating that neither specific type of detail uniquely predicted variance, but events that were closer in time were associated with a stronger perception of perceived control and intention to engage in the future event.

**Table 3:** Summary of regression analyses of content and spatial details on future thinking variables (all* N* = 54)

DV	Overall detail/Vividness			Mental imagery			Pre-experiencing			Anticipated pleasure			Anticipatory pleasure			Perceived control			Intention		
	*B*	SE* B*	*β*	*B*	SE* B*	*Β*	*B*	SE* B*	*β*	*B*	SE* B*	*β*	*B*	SE* B*	*β*	*B*	SE* B*	β	*B*	SE* B*	β
Spatial Details	0.85	0.19	0.59***	0.77	0.21	0.56**	0.73	0.16	0.56***	0.44	0.20	0.40*	0.50	0.19	0.44*	0.03	0.26	0.02	– 0.16	0.19	– 0.15
Content Details	– 0.07	0.20	– 0.04	– 0.04	0.22	– 0.02	– 0.02	0.17	0.– 01	– 0.34	0.21	– 0.29	– 0.25	20	– 0.20	0.29	0.27	0.13	0.13	0.20	0.12
Temporal Distance	0.34	– 19	– 0.19†	0.13	0.21	0.08	– 0.23	0.16	– 0.14	0.14	0.20	0.10	0.01	0.19	0.01	– 0.73	0.26	– 0.36**	– 0.52	0.19	– 0.38**
Age	0.03	0.02	0.17	0.02	0.02	0.14	0.02	0.01	0.15	0.00	0.02	0.06	0.03	0.02	0.23	0.04	0.02	0.19	0.01	0.02	0.10
Gender	0.27	0.29	0.09	0.17	0.33	0.06	0.56	0.26	0.21*	0.06	0.31	0.02	0.43	0.30	0.16	0.42	0.40	0.13	0.22	0.20	0.10
*F*			110.1***			50.6***			120.1***			10.1			30.5**			30.9**			10.8
*R*^*2*^			0.54			0.37			0.56			0.11			0.27			0.29			0.16
Adjusted *R*^*2*^			0.49			0.30			0.51			0.01			0.19			0.21**			0.07

*DV* dependent variable in the regression model

^†^*p* < 0.10, *p < 0.05, **p < 0.01, ***p < 0.001

### Aim 2: manipulation checks

A 3 (condition: baseline, content, spatial) × 2 (detail type: content, spatial) ANOVA indicated a main effect for condition, *F*(2, 106) = 22.2, *p* < 0.001, $\eta_{{\text{p}}}^{2}$ = 0.22, no main effect for detail type, *F*(1, 53) = 1.3, *p* = 0.245, $\eta_{{\text{p}}}^{2}$ = 0.02, and a condition by detail type interaction effect, *F*(2, 106) = 4.6, *p* = 0.011, $\eta_{{\text{p}}}^{2}$ = 0.81. Follow-up tests for *content* details indicated participants reported higher detail in the content condition relative to baseline (*M* difference = 0.41, 95% CI 0.16, 0.67; *t*(53) = 3.3, *p* = 0.002, *d*_*z*_ = 0.45), and in the spatial condition relative to baseline (*M* difference = 0.48, 95% CI 0.21, 0.75; *t*(53) = 3.5, *p* = 0.001, *d*_*z*_ = 0.49), but there was no significant difference between content and spatial conditions (*M* difference = 0.06, 95% CI − 0.10, 0.23; *t*(53) = 0.8, *p* = 0.426, *d*_*z*_ = 0.11). Follow-up tests for *spatial* details indicated participants reported higher detail in the content condition relative to baseline (*M* difference = 0.60, 95% CI 0.32, 0.89; *t*(53) = 4.2, *p* < 0.001, *d*_*z*_ = 0.57), and in the spatial condition relative to baseline (*M* difference = 0.87, 95% CI 0.60, 1.13; *t*(53) = 6.6, *p* < 0.001, *d*_*z*_ = 0.90). Spatial details were reported as significantly higher in the spatial condition relative to the content condition (*M* difference = 0.26, 95% CI − 0.04, 0.48; *t*(53) = 2.3, *p* = 0.021, *d*_*z*_ = 0.33). In summary, content details increased in both conditions from baseline to a similar degree, whereas spatial details increased in both conditions and were higher in the spatial condition relative to the content condition.

### Aim 2: repeated measures analyses

Firstly, effects of the guided future thinking conditions were assessed on overall detail/vividness and mental imagery. A repeated measures ANOVA for detail/vividness showed a main effect for condition, *F*(2, 106) = 18.1, *p* < 0.001, $\eta_{{\text{p}}}^{2}$ = 0.25. Participants reported higher detail/vividness in the content condition relative to baseline (*M* difference = 0.61, 95% CI 0.25, 0.98; *t*(53) = 3.3, *p* = 0.001, *d*_*z*_ = 0.45), and in the spatial condition relative to baseline (*M* difference = 0.93, 95% CI 0.63, 1.23; *t*(53) = 6.2, *p* < 0.001, *d*_*z*_ = 0.85), and overall detail/vividness was significantly higher in the spatial condition relative to the content condition (*M* difference = 0.31, 95% CI 0.03, 0.59; *t*(53) = 2.2, *p* = 0.028, *d*_*z*_ = 0.30).

A main effect was also found for mental imagery, *F*(2, 62) = 18.9, *p* < 0.001, $\eta_{{\text{p}}}^{2}$ = 0.26. More use of mental imagery was reported in the content condition relative to baseline (*M* difference = 0.71, 95% CI 0.37, 1.05; *t*(53) = 4.1, *p* < 0.001, *d*_*z*_ = 0.56), and in the spatial condition relative to baseline (*M* difference = 0.96, 95% CI 0.60, 1.32; *t*(53) = 5.3, *p* < 0.001, *d*_*z*_ = 0.72), but the trend for a difference between content and spatial conditions did not reach statistical significance (*M* difference = 0.25, 95% CI 0.01, 0.52; *t*(53) = 1.7, *p* = 0.068, *d*_*z*_ = 0.25).

A main effect was found for pre-experiencing, *F*(2, 106) = 15.5, *p* < 0.001, $\eta_{{\text{p}}}^{2}$ = 0.22. Participants reported stronger pre-experiencing in the content condition relative to baseline (*M* difference = 0.58, 95% CI 0.28, 0.88; *t*(53) = 3.9, *p* < 0.001, *d*_*z*_ = 0.53), and in the spatial condition relative to baseline (*M* difference = 0.66, 95% CI 0.39, 0.94; *t*(53) = 4.8, *p* < 0.001, *d*_*z*_ = 0.66), but the difference between content and spatial conditions was not significant (*M* difference = 0.08, 95% CI − 0.11, 0.28; *t*(53) = 0.8, *p* = 0.419, *d*_*z*_ = 0.10).

Next, the anticipated and anticipatory pleasure items were assessed for change over the conditions. A main effect was found for anticipated pleasure, *F*(2, 106) = 3.2, *p* = 0.042, $\eta_{{\text{p}}}^{2}$ = 0.05. Participants reported lower anticipated pleasure in the content condition relative to baseline (*M* difference = 0.24, 95% CI − 0.01, − 0.47; *t*(53) = 2.0, *p* = 0.041, *d*_*z*_ = 0.24), but no significant difference in the spatial condition relative to baseline (*M* difference = 0.20, 95% CI − 0.00, − 0.41; *t*(53) = 1.9, *p* = 0.058, *d*_*z*_ = 0.26). The difference between content and spatial conditions was not significant (*M* difference = 0.03, 95% CI − 0.08, 0.20; *t*(53) = 0.4, *p* = 0.649, *d*_*z*_ = 0.00). No main effect for anticipatory pleasure, *F*(2, 106) = 2.2, *p* = 0.111, $\eta_{{\text{p}}}^{2}$ = 0.04, indicating conditions did not significantly differ.

A main effect was found for perceived control, *F*(2, 106) = 10.6, *p* < 0.001, $\eta_{{\text{p}}}^{2}$0.16. Participants reported higher perceived control in the content condition relative to baseline (*M* difference = 0.46, 95% CI 0.01, 0.91; *t*(53) = 2.0, *p* = 0.045, *d*_*z*_ = 0.31), and in the spatial condition relative to baseline (*M* difference = 0.88, 95% CI 0.51, 1.2; *t*(53) = 4.8, *p* < 0.001, *d*_*z*_ = 0.70), and perceived control was significantly higher in the spatial condition relative to the baseline condition (*M* difference = 0.42, 95% CI 0.10, 0.74; *t*(53) = 2.6, *p* = 0.011, *d*_*z*_ = 0.36). No main effect for condition was found for behavioural intention, *F*(2, 106) = 0.4, *p* = 0.631, $\eta_{{\text{p}}}^{2}$ = 0.00, indicating conditions did not significantly differ.

As requested by a reviewer, to assess if there were repetition suppression effects or whether participants improved purely as a function of repeated guided simulation, regardless of condition, *t-*tests were conducted across the three conditions in the order they were presented for participants. Some condition order information was not available due to a clerical error, leaving *n* = 39 for these analyses. The results did not show evidence of either of these effects, with guided interview conditions both being higher on content, spatial, overall detail/vividness, and perceived control than baseline (all *p*’s < 0.05), and no difference between the first and second guided interviews (all *p*’s > 0.05). Consistent with the results above, for anticipated and anticipatory pleasure and behavioural intention, the guided interview conditions did not differ from baseline, nor the first and seconded guided interviews differ from each other (all *p*’s > 0.05).

## Discussion

Overall, these findings are consistent with previous studies showing effortful simulations of autobiographical events increase their detail and vividness (e.g., Boland et al., 2018; Szpunar & Schacter, 2013) and mental imagery (Blackwell et al., 2015). Extending on Rubin et al.’s (2019) work in autobiographical memory, the cross-sectional findings indicate spatial details for future thinking were a unique predictor of overall detail and vividness, pre-experiencing, mental imagery, and anticipatory pleasure. Spatial details were also associated with anticipated pleasure in regression analyses. This indicates that spatial details are a key factor in imagining vivid, visually rich events that are strongly pre-experienced and evoke positive emotional responses. For perceived control and behavioural intention, only temporal distance was found to be a unique, significant predictor in the regression model. Therefore, how far into the future an event was perceived to occur was the dominant factor in how much it was under one’s control and intended to be enacted. This is consistent with findings that events further in time are perceived as being less realistic (D’Argembeau & Van der Linden, 2004).

Content and spatial details increased after both simulation conditions. This is likely a result of the repeated simulation of the events from baseline to the guided simulation conditions, which is known to increase such phenomenological details (see Szpunar & Schacter, 2013 for a demonstration of this). However, there were differences between the two simulation conditions which cannot be attributed to mere repetition of event simulation, given that the order of these conditions was counterbalanced. Spatial details were significantly higher in the spatial condition, however, content details were not significantly higher in the content condition. This indicates that focusing on spatial details increases these details from baseline, as expected, but might also causes an increase in content details to a similar degree to a condition in which they are specifically focused on. This is consistent with predictions of scene construction theory and related findings (Rubin & Umanath, 2015; Sheldon et al., 2019). Given this, differences between the simulation conditions on other variables might be attributable to an increased focus on spatial details rather than a decreased, or relatively decreased, focus on content details. Indeed, as indicated in the cross-sectional analyses, content details do not seem to uniquely predict future thinking characteristics. These findings suggest that, (a) it may be difficult to increase content details as distinct from spatial details, and (b) focusing on spatial details specifically may have effects separate from focusing on content details. The findings also suggest that outside of the laboratory when people construct mental scenes of future events, this process will give rise to content details despite not purposively elaborating on them. Although we and other researchers (Sheldon et al., 2019) have now found some selective effects of focusing attention onto spatial details, research that is more naturalistic such as diary or ecological momentary assessment designs, would help to identify if and how this occurs in daily life.

Both conditions increased in overall detail and vividness, mental imagery, and pre-experiencing. However, focusing on spatial details produced more overall detail and vividness relative to the content condition, with differences in imagery trending in the expected direction. These findings again support scene construction theory in placing spatial contextual details in a position of importance in generating rich mental representations of autobiographical events (Hassabis & Maguire, 2007; Rubin & Umanath, 2015; Rubin et al., 2019). However, the lack of difference between conditions in pre-experiencing was somewhat inconsistent with this. Given the average score on spatial details was only approximately five out of seven following the guided future thinking, more elaboration or integration of other aspects of the future event may be needed to elicit the sense of pre-experiencing. Further investigation is required.

The direction of change for anticipated and anticipatory pleasure was unexpected, and contrary to previous findings on affective responses to future events (Boland et al., 2018; Hallford et al., 2020b; Holmes et al., 2008, 2009; Quoidbach et al., 2009). This may be due to the guided future thinking focusing on particular details of the future event (e.g., room details, object details) rather than positive feelings. In addition to details, general appraisals of a future event and an incubation of how enjoyable a future event might be needed to elicit an affective response. Indeed, large effects have been observed on anticipated and anticipatory pleasure when guided future thinking focused on imagining aspects related to pleasure or enjoyment (Hallford et al., 2020b).

Perceived control increased in both conditions, and more so in the spatial condition. This supports previous findings that detailed and vivid future thinking is associated with increases in perceived control over future events (Boland et al., 2018; Brown et al., 2002; Jing et al., 2016), and extends on them by showing that spatial features further increase perceived control. No significant change was found on behavioural intention, despite previous findings on intention and perceived likelihood of occurrence (Boland et al., 2018; Brown et al., 2002; Hallford et al., 2020b). As above, this may be attributed to the lack of focus on enjoyment from experiencing the future event, which is a strong motivating factor in behavioural intention (Bagozzi & Pieters, 1998; Baumgartner et al., 2008) and decision-making (Engel et al., 2013; Mellers et al., 1999; Sherdell et al., 2012).

The results may have implications for interventions and treatments that leverage guided future thinking to treat mental health issues. A great number of evidence-based cognitive-behavioural therapies use future thinking, for example, in order to increase rewarding behaviours (e.g., in activity scheduling), solve-problems, and generate personal goals. Incorporating a focus on spatial details when imagining future events might incrementally improve generation of detailed and vivid thoughts and increase perceived control. In particular, this might have relevance for disorders characterised by impairments in episodic future thinking, such as in major depression (Hallford et al., 2020a) and schizophrenia-spectrum disorders (Hallford et al., 2018). Further testing to establish these selective effects in clinical groups is needed though. The findings are also relevant for interventions focused on improving autobiographical thinking, such as Future Specificity Training (FeST; Hallford et al., 2020d). Incorporating elaboration on spatial details in these programs might improve the effects on outcomes such as use of detail/vividness and perceived control. In particular, a sense of control and competence over future events has relevance for a range of emotional disorders and is generally considered a modifiable factor in mental health and interventions (Barlow et al., 2017). Although out of the scope of this study, future research might assess whether these selective effects also occur for negatively-valenced events. Particularly for interventions that aim to increase self-efficacy or control over future events. Simulating solutions to worrying future events in more detail has been associated with increased perceived control and reduced worry about them (Jing et al., 2016). Including a focus on spatial details may help counter negative expectations of outcomes.

A limitation of this study is that ratings for the events were directly provided to the experimenters, potentially introducing a response bias or demand characteristics, and inflating the ratings of future events. This may be the case despite participants being encouraged to provide their ratings honestly. Participants were not aware of the specific hypothesis of spatial details leading to higher scores though, and therefore we can likely rule out demand characteristics relating to differences between guided future thinking conditions. In future, having participants write their responses might help partially mitigate biases. Assessing details using objective measures, such as in Sheldon et al. (2019) would also provide another useful variable. Although this would not eliminate possible biases, it could be contrasted with subjective ratings and coded for detail. The reliability of some of the questions pertaining to future events, in particular anticipatory pleasure, could be improved by introducing multi-item measures of these constructs. Another consideration is to examine familiarity with the spatial location of simulated future events, given this has been shown to be associated with reported vividness, with nearer events with more familiar locations (Arnold et al., 2011). While not assessed in this study, familiarity of location may be an important moderator, in conjunction with temporal distance, to assess in future studies. In conclusion, this study provides evidence that there are selective effects of focusing on spatial details, relative to content, when engaging in future thinking for positive events.

## Data Availability

Upon request from the corresponding author.

## Code availability

Upon request from the corresponding author.
